# Van Neck–Odelberg Disease in a Pediatric Patient With Prolonged Groin Pain: A Case Report

**DOI:** 10.1155/crra/6687686

**Published:** 2026-10-31

**Authors:** Biswajit Borborah

**Affiliations:** ^1^ Queen Elizabeth the Queen Mother Hospital Margate UK nhs.uk

**Keywords:** case report, groin pain, ischiopubic synchondrosis, magnetic resonance imaging, Van Neck–Odelberg disease

## Abstract

Van Neck–Odelberg disease is a benign disorder of the ischiopubic synchondrosis (IPS) that can be mistaken for infection, fracture, or bone tumor. A boy aged 10 years and 7 months presented with a 1‐year history of exertional groin pain and intermittent limping. The pain was bilateral but consistently worse on the right and was aggravated by football and running. He was afebrile. Examination showed a mild right antalgic gait, focal tenderness over the right ischiopubic region, and pain on combined right hip flexion and external rotation. The white‐cell count (4.9 × 10^9^/L) and erythrocyte sedimentation rate (2 mm/h) were within the reference ranges. A C‐reactive protein result was unavailable. Pelvic radiography showed smooth, asymmetric enlargement and central lucency of the right IPS without an aggressive periosteal reaction. MRI confirmed widening of the synchondrosis with surrounding marrow edema. The cortex remained intact, and there was no abscess or soft‐tissue mass. A transverse hypointense band perpendicular to the pubic axis represented the characteristic fibrous bridge. The clinical and imaging findings supported a diagnosis of symptomatic Van Neck–Odelberg disease, and biopsy was avoided. Management consisted of acetaminophen as required, activity restriction for 2 weeks, and once‐weekly physiotherapy for 6 months with hip adductor and external rotator stretching. Initial improvement in pain and limping occurred at 4–6 weeks, with complete clinical recovery at 6 months. The combination of a 1‐year symptom history, bilateral pain with unilateral imaging findings, and a preserved fibrous bridge illustrates how recognition of the typical imaging appearance, together with the clinical and laboratory findings, can prevent unnecessary antibiotics, biopsy, and oncological investigation.

**Learning Points**

• The ischiopubic synchondrosis (IPS) is a normal temporary joint; asymmetric enlargement may be symptomatic and can mimic infection, fracture, or tumor.
• Marrow edema on MRI is nonspecific. An intact cortex and a preserved transverse fibrous bridge are reassuring features when clinical and laboratory findings do not suggest infection.
• Foot dominance should be documented rather than inferred from the affected side.
• Conservative management is appropriate when aggressive features are absent, with clinical reassessment if symptoms worsen or fail to resolve.

## 1. Introduction

The IPS is a temporary cartilaginous junction between the inferior pubic ramus and the ischium. It becomes progressively thinner and usually fuses before puberty. Enlargement may be bilateral in younger children and asymmetric later in childhood. When the enlarged synchondrosis is painful, the condition is known as Van Neck–Odelberg disease or symptomatic osteochondrosis of the IPS [1–3].

The radiographic lucency and expansion of the IPS, particularly when accompanied by marrow edema on MRI, can resemble osteomyelitis, a stress fracture, or a bone tumor. Familiarity with the nonaggressive appearance of the condition can help avoid invasive investigations and unnecessary treatment.

We report a physically active boy with a 1‐year history of bilateral activity‐related groin pain but unilateral right‐sided IPS enlargement. The educational value lies in the combination of prolonged symptoms, discordance between symptom laterality and imaging findings, and a visible transverse fibrous bridge on MRI. These features illustrate the importance of correlating the site of tenderness with nonaggressive imaging findings before considering invasive investigation. This case report was prepared in accordance with the CARE reporting guidelines; an accompanying checklist identifies the reported items and information that is unavailable.

## 2. Case Description

### 2.1. Clinical History and Examination

A boy aged 10 years and 7 months presented with bilateral groin pain and intermittent limping of approximately 1 year′s duration. The symptoms were consistently worse on the right. He played football frequently, and both running and football aggravated the pain; there was little or no pain at rest. There was no history of acute trauma, fever, weight loss, or other systemic symptoms. His medical, family, developmental, and psychosocial histories were unremarkable. The dominant kicking leg was not recorded.

He walked with a mild right antalgic gait. There was focal tenderness over the right ischiopubic region. Hip flexion was approximately 100° bilaterally and was painless when tested alone, although pain was reproduced with combined flexion and external rotation of the right hip. Internal and external rotation measured approximately 60° and 40°, respectively, on both sides. Log‐roll and telescoping tests were negative.

### 2.2. Laboratory Assessment

The white‐cell count was 4.9 × 10^9^/L (reference range, 4.0–11.0 × 10^9^/L), and the erythrocyte sedimentation rate was 2 mm/h (reference range, 1–10 mm/h). Both were within the stated reference ranges. A C‐reactive protein (CRP) result was unavailable.

### 2.3. Imaging Assessment

An anteroposterior pelvic radiograph showed smooth asymmetric enlargement of the right IPS with a few central lucent areas. There was no cortical destruction, aggressive periosteal reaction, or pathological fracture (Figure 1).

**Figure 1:**
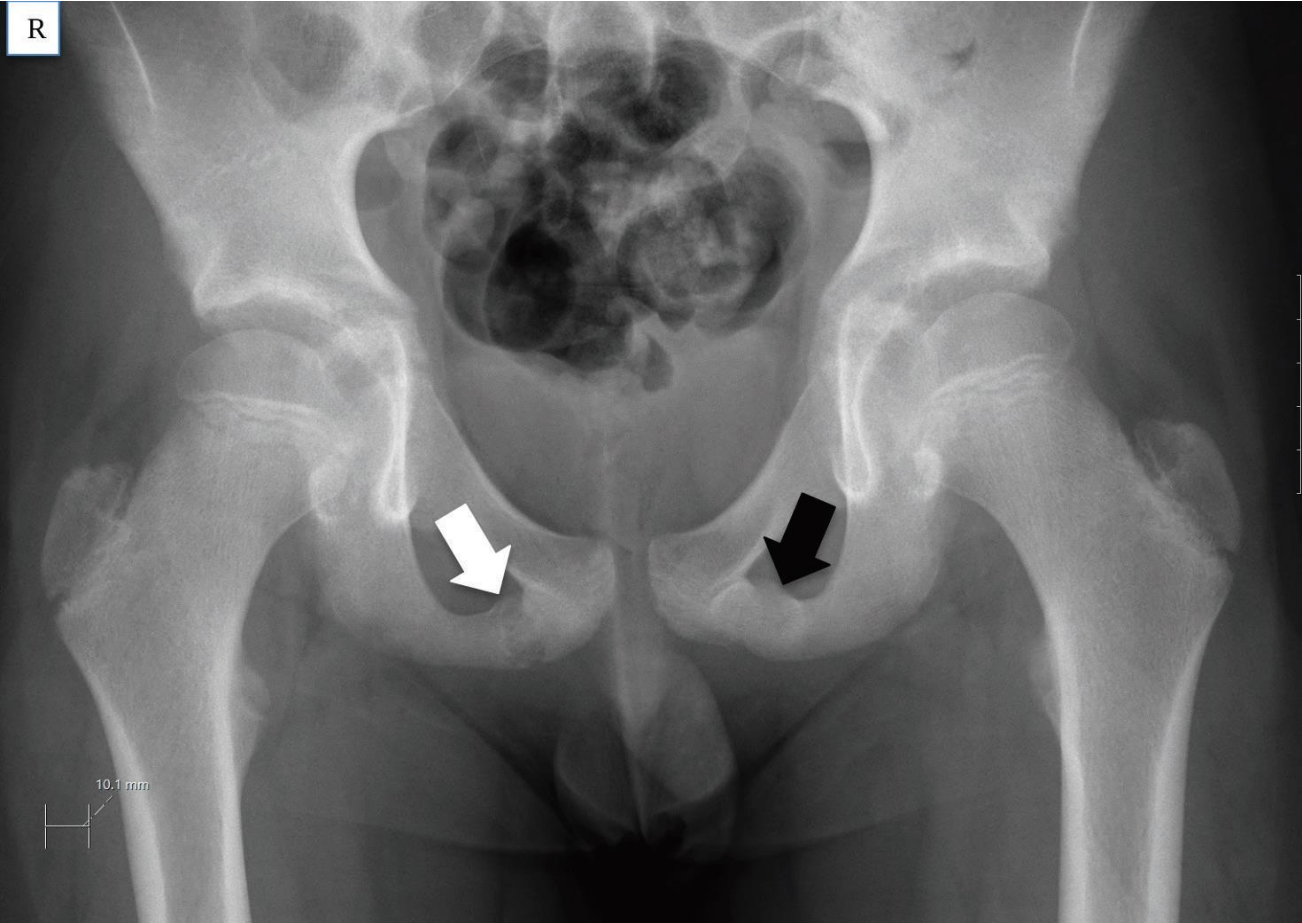
Anteroposterior pelvic radiograph. The white arrow indicates smooth enlargement and central lucency of the right ischiopubic synchondrosis; the black arrow indicates the left ischiopubic synchondrosis for comparison. R, right.

MRI of the pelvis was performed on a 1.5‐T Philips scanner. The protocol included coronal short‐tau inversion recovery and T1‐weighted turbo spin‐echo sequences, together with axial T1‐ and T2‐weighted turbo spin‐echo sequences. Intravenous contrast was not administered.

MRI showed widening of the right IPS with surrounding marrow edema. Centrally, a smooth transverse hypointense band ran perpendicular to the pubic axis, consistent with fibrous bridging across the synchondrosis, most clearly demonstrated on the axial T2‐weighted image (Figures 2, 3, and 4). The cortex was intact. There was no aggressive marrow‐replacing lesion, periosteal reaction, adjacent soft‐tissue mass, myositis, fluid collection, or abscess.

**Figure 2:**
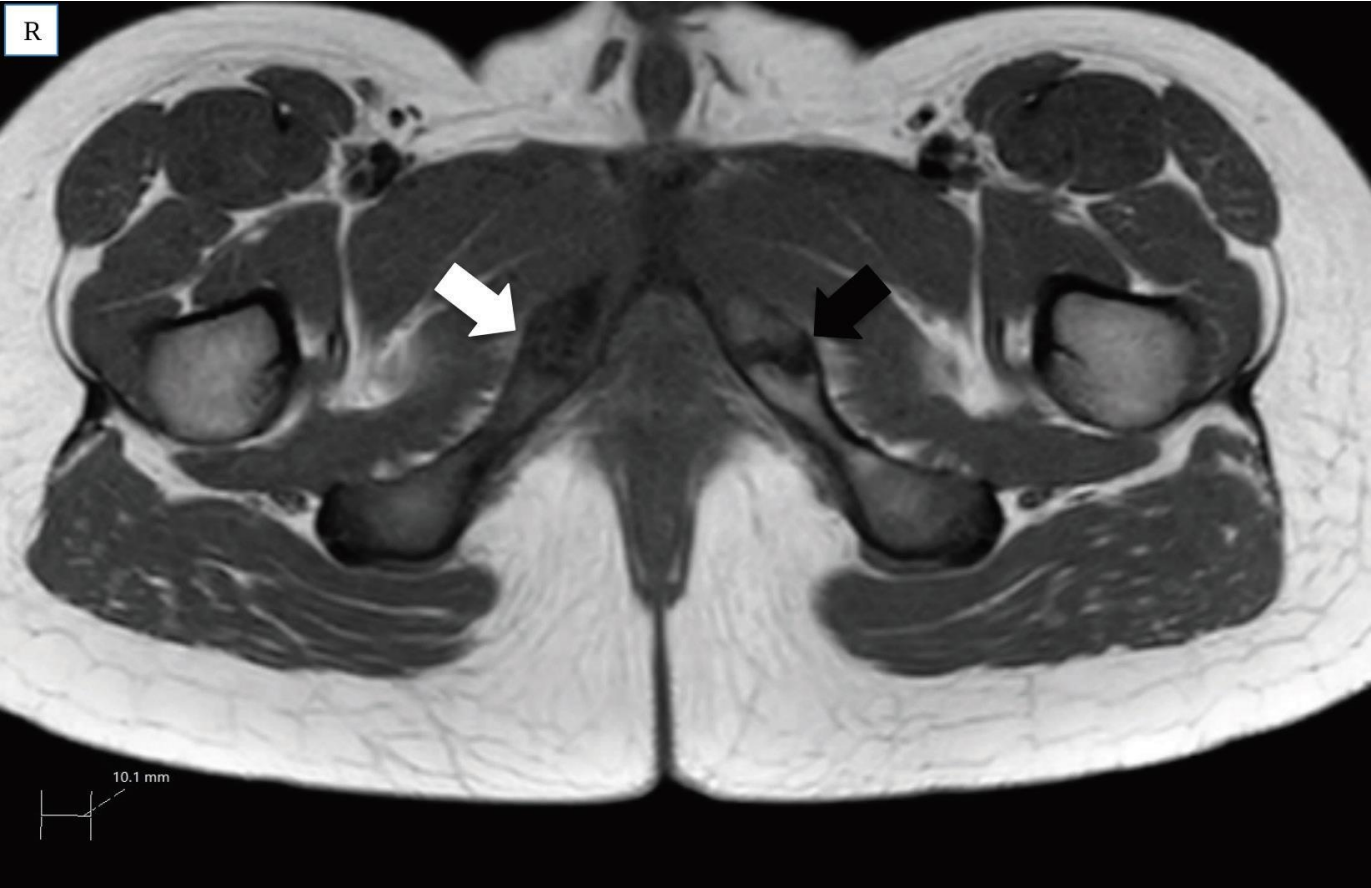
Axial T1‐weighted magnetic resonance image. The white arrow indicates the enlarged right ischiopubic synchondrosis; the black arrow indicates the left ischiopubic synchondrosis for comparison. R, right.

**Figure 3:**
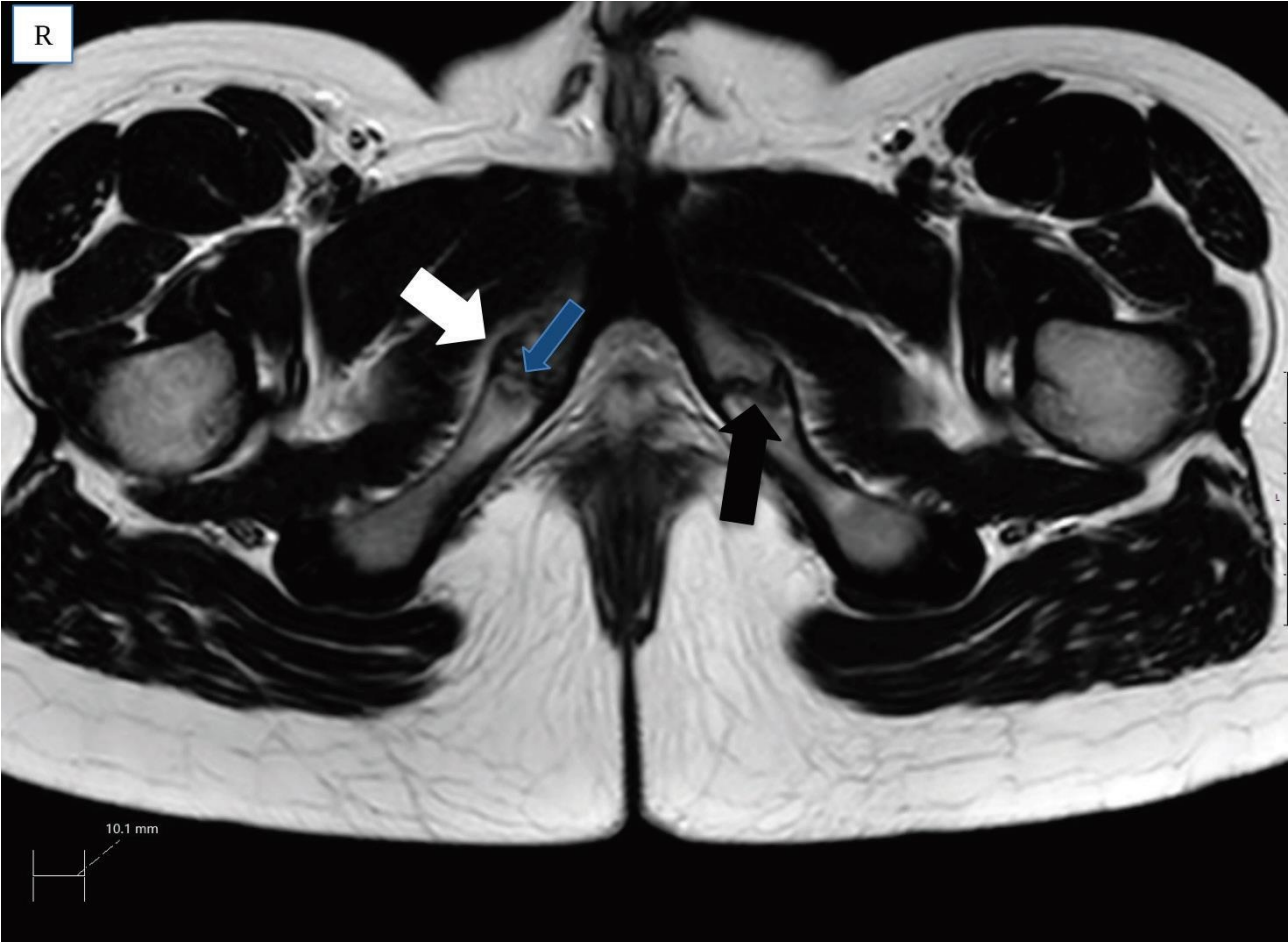
Axial T2‐weighted magnetic resonance image. The white arrow indicates the enlarged right ischiopubic synchondrosis, the blue arrow indicates the transverse low‐signal fibrous bridge, and the black arrow indicates the left ischiopubic synchondrosis. R, right.

**Figure 4:**
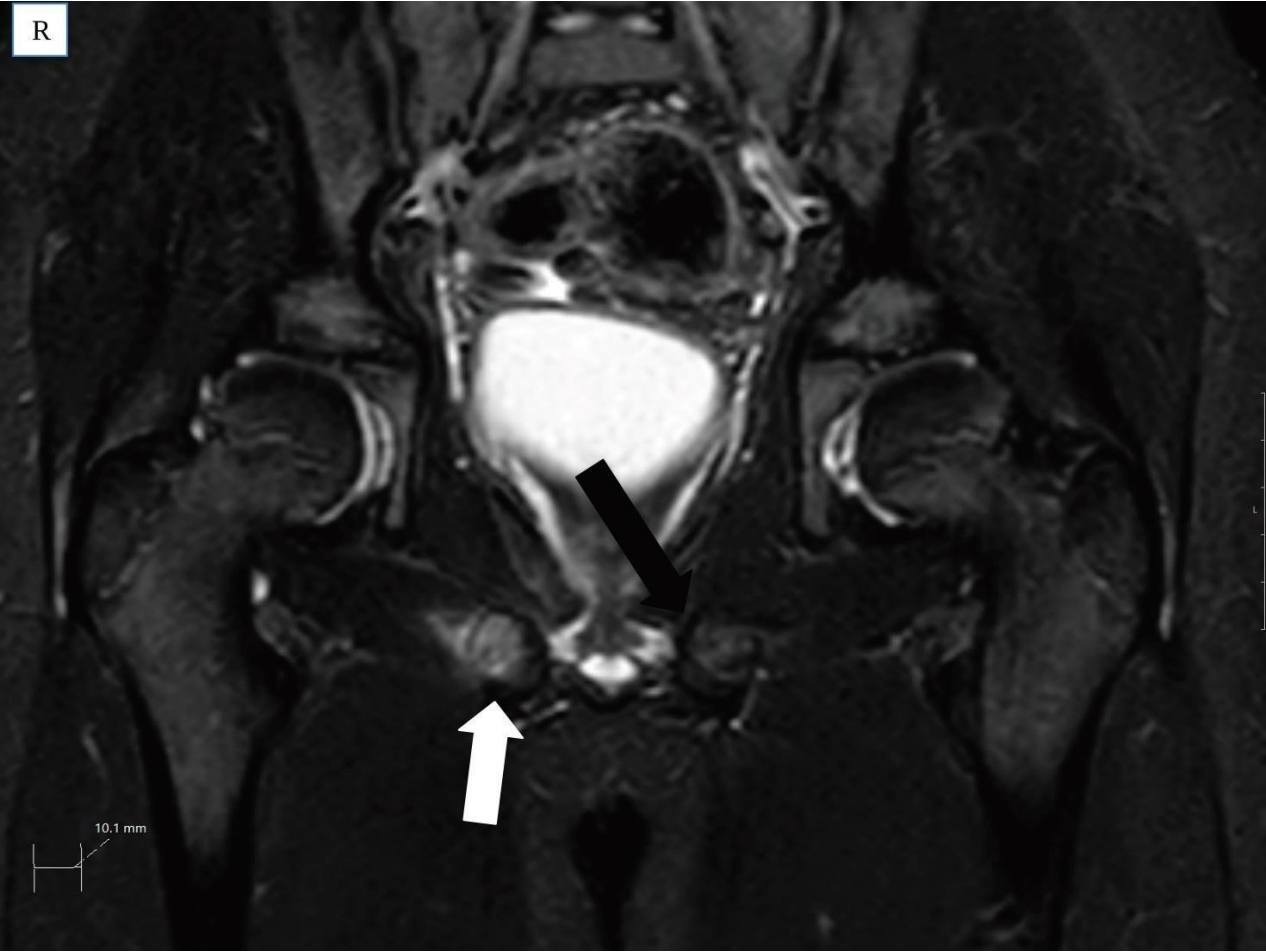
Coronal short‐tau inversion recovery magnetic resonance image. The white arrow indicates marrow edema at the right ischiopubic synchondrosis; the black arrow indicates the left ischiopubic synchondrosis for comparison. R, right.

### 2.4. Diagnostic Reasoning and Final Diagnosis

The findings were considered diagnostic of symptomatic Van Neck–Odelberg disease of the right IPS. The patient′s age, activity‐related pain, focal tenderness, and smooth enlargement of the synchondrosis were typical. Marrow edema was centered on the IPS, whereas the cortex was preserved, and the central fibrous bridge remained visible on axial T2‐weighted images. The normal white‐cell count and erythrocyte sedimentation rate further reduced the likelihood of infection.

Osteomyelitis was considered because it can also produce pain and marrow edema at the IPS. However, the patient had no fever, leucocytosis, elevated erythrocyte sedimentation rate, cortical destruction, adjacent myositis, or collection. Ewing sarcoma was unlikely in the absence of an aggressive marrow lesion, interrupted cortex, aggressive periosteal reaction, or soft‐tissue mass. A stress fracture would be expected to show a fracture line, sometimes with cortical disruption or periosteal reaction; the low‐signal line in this case had the position and orientation of the normal fibrous bridge. Although asymmetric IPS closure may be incidental, the focal tenderness, right‐predominant pain, and local marrow edema indicated that the finding was symptomatic. Biopsy was therefore not performed.

### 2.5. Treatment

The patient and his family were reassured that the condition was benign and self‐limiting. He was given acetaminophen as required for pain and advised to restrict activity for 2 weeks. The prescribed dose was not documented. Physiotherapy was then undertaken once a week for 6 months and focused on stretching the hip adductor and external rotator muscles.

### 2.6. Follow‐Up and Outcome

Initial improvement in pain and limping occurred at 4–6 weeks after starting conservative management. Physiotherapy continued once weekly for 6 months, and complete clinical recovery was documented at 6 months. Because analgesia, activity modification, and physiotherapy were used together, their individual contributions to recovery could not be determined. Follow‐up imaging was not obtained because the child had recovered clinically, and the initial imaging showed no concerning features.

The family was advised that the radiographic abnormality could remain visible during skeletal maturation even after the symptoms had settled. They were asked to seek reassessment if the pain worsened, systemic symptoms developed, or function deteriorated.

### 2.7. Patient Timeline

The timeline of symptoms, diagnostic assessment, treatment, and clinical recovery is summarized in Table 1.

**Table 1:** Clinical course using relative time points.

Time point	Clinical events
Approximately 12 months before presentation	Bilateral activity‐related groin pain and intermittent limping began; symptoms were consistently worse on the right.
At presentation, aged 10 years and 7 months	Right antalgic gait and focal right ischiopubic tenderness. White‐cell count and erythrocyte sedimentation rate were normal; CRP result unavailable.
Diagnostic assessment	Radiography and MRI showed right IPS enlargement, marrow edema, and a preserved fibrous bridge without aggressive features. Symptomatic Van Neck–Odelberg disease was diagnosed.
Initial treatment	Acetaminophen as required and activity restriction for 2 weeks; once‐weekly physiotherapy subsequently continued for 6 months.
4–6 weeks after starting treatment	Initial improvement in pain and limping.
6 months	Complete clinical recovery. No follow‐up imaging was obtained.

## 3. Discussion

Van Neck–Odelberg disease reflects delayed ossification and symptomatic enlargement of a normal developmental structure. Repetitive traction from the hamstring and adductor muscles, together with asymmetric loading across the pelvis, has been proposed as a reason for delayed fusion and pain [1, 2, 4, 5]. In this child, frequent football and the clear relationship between running and pain make mechanical loading a plausible contributor, although causation cannot be established from a single case.

The age and imaging findings in this case are similar to those reported in the literature. A 2024 systematic review identified 43 individual cases, including 28 boys and 15 girls [6]. Schneider et al. [7] reported a median age of 10 years in their 21‐patient cohort, and 17 patients had unilateral disease. Our patient also had unilateral imaging changes, but his pain was bilateral and more marked on the right. No corresponding marrow edema or aggressive abnormality was seen on the left. Altered gait or referred pain could explain the left‐sided symptoms, but this remains speculative. Bilateral pain should therefore not be equated with bilateral imaging‐confirmed disease.

Sport‐related symptoms have been described in several reports. McLoughlin et al. [8] described a 9‐year‐old competitive gymnast with right hip pain, and Fonseca et al. [5] reported a 6‐year‐old futsal player whose mechanical groin pain was managed by stopping sport. Sorce and Fumière [9] reported improvement after 1 month of anti‐inflammatory treatment and cessation of sport in a 10‐year‐old gymnast and swimmer. Our patient showed initial improvement at 4–6 weeks, with complete clinical recovery at 6 months. Initial symptom improvement and complete recovery are distinct outcomes and should not be used interchangeably. In the larger series by Schneider et al. [7], the median time to return to physical activity was 5 months. Differences in symptom severity, treatment, and the definition of recovery may account for the variation.

The suggested relationship between IPS asymmetry and foot dominance should be applied cautiously. Herneth et al. [2] studied 32 children; among the nine with unilateral enlargement, all seven with left‐sided enlargement were right‐footed, whereas both children with right‐sided enlargement were left‐footed. These findings support delayed fusion on the weight‐bearing, nondominant side, but the subgroup was small. As foot dominance was not documented in our patient, no such association can be made in this case.

MRI findings must be interpreted alongside the history, examination, and inflammatory markers. Marrow edema is not specific and may also occur with infection or stress injury. Smooth IPS enlargement, intact cortical margins, absence of an aggressive periosteal reaction or soft‐tissue mass, and preservation of the central low‐signal fibrous bridge favor Van Neck–Odelberg disease [3, 10]. Fever, raised inflammatory markers, extensive soft‐tissue inflammation, an abscess, cortical destruction, or an aggressive mass should instead prompt investigation for osteomyelitis or neoplasm [1, 4, 9]. In our patient, the preserved fibrous bridge and cortex, absence of aggressive soft‐tissue findings, normal white‐cell count, and normal erythrocyte sedimentation rate supported conservative management without biopsy or antibiotic treatment. The CRP result was unavailable, and normal available laboratory results alone do not exclude infection.

There are several limitations. A CRP result was unavailable, limiting the laboratory assessment of inflammation. Foot dominance and the acetaminophen dose were not documented. The report does not include a separate patient or parent perspective, formal adherence or tolerability measures, or a systematic assessment of adverse events. Histological confirmation was not obtained, as biopsy was not justified for a benign‐appearing, self‐limiting condition. No follow‐up MRI was performed, so the outcome was assessed clinically rather than radiologically. Follow‐up was limited to 6 months, and later recurrence cannot be excluded. Finally, medication, activity restriction, and physiotherapy were introduced during the same period, making it impossible to determine which intervention contributed most to recovery.

## 4. Conclusion

Van Neck–Odelberg disease is an important consideration in a school‐aged child with atraumatic, activity‐related groin pain and limping. Smooth enlargement of the IPS, surrounding marrow edema, and a preserved transverse fibrous bridge support the diagnosis when the cortex is intact and there is no abscess, soft‐tissue mass, or clinical evidence of systemic illness. Recognizing this pattern allows conservative management and avoids unnecessary antibiotics, biopsy, and oncological investigation.

## Funding

No funding was received for this manuscript.

## Ethics Statement

This case report was prepared using anonymized patient data. In accordance with institutional policy, formal research ethics approval was not required for the publication of a single, anonymized case report.

## Consent

Written informed consent for publication of the clinical details and images was obtained from the patient′s parent or legal guardian. The patient′s assent was obtained where appropriate. All clinical information and images were anonymized before publication.

## Conflicts of Interest

The author declares no conflicts of interest.

## Data Availability

The data that support the findings of this study are available on request from the corresponding author. The data are not publicly available due to privacy or ethical restrictions.
